# Azole resistance among clinical *Aspergillus fumigatus* species complex isolates in Greece: first detection of TR_46_-mediated resistance and azole-resistant cryptic species

**DOI:** 10.1093/jacamr/dlag238

**Published:** 2026-11-02

**Authors:** Maria Siopi, Margarita Vargiami, Maria-Ioanna Beredaki, Aristea Velegraki, Levantia Zachariadou, Emmanuel Roilides, Georgios Chamilos, Stavroula Antonopoulou, Lemonia Skoura, Ana Alastruey-Izquierdo, Spyros Pournaras, Joseph Meletiadis

**Affiliations:** Clinical Microbiology Laboratory, ‘Attikon’ University General Hospital, Medical School, National and Kapodistrian University of Athens, Athens, Greece; Clinical Microbiology Laboratory, ‘Attikon’ University General Hospital, Medical School, National and Kapodistrian University of Athens, Athens, Greece; Clinical Microbiology Laboratory, ‘Attikon’ University General Hospital, Medical School, National and Kapodistrian University of Athens, Athens, Greece; Department of Microbiology, School of Medicine, National and Kapodistrian University of Athens, Athens, Greece; Department of Microbiology, ‘Aghia Sophia’ Children’s Hospital, Athens, Greece; Infectious Diseases Unit, 3^rd^ Department of Pediatrics, Aristotle University School of Medicine and ‘Hippokration’ General Hospital, Thessaloniki, Greece; Laboratory of Clinical Microbiology and Microbial Pathogenesis, School of Medicine, University of Crete, Heraklion, Crete, Greece; Department of Clinical Microbiology, ‘Alexandra’ General Hospital, Athens, Greece; Department of Microbiology, ‘AHEPA’ University Hospital, School of Medicine, Aristotle University of Thessaloniki, Thessaloniki, Greece; Mycology Reference Laboratory, National Centre for Microbiology, Instituto de Salud Carlos III, Majadahonda, Madrid, Spain; Center for Biomedical Research in Network in Infectious Diseases (CIBERINFEC), Instituto de Salud Carlos III, Madrid, Spain; Clinical Microbiology Laboratory, ‘Attikon’ University General Hospital, Medical School, National and Kapodistrian University of Athens, Athens, Greece; Clinical Microbiology Laboratory, ‘Attikon’ University General Hospital, Medical School, National and Kapodistrian University of Athens, Athens, Greece

## Abstract

**Objectives:**

To assess the prevalence of antifungal resistance among clinical *Aspergillus fumigatus* species complex (SC) isolates in Greece, focusing on azole resistance and its underlying molecular mechanisms.

**Methods:**

A total of 249 clinical *A. fumigatus* SC isolates were collected from geographically distinct centres across Greece. Antifungal susceptibility testing (AFST) was conducted according to EUCAST guidelines. Cryptic species and azole-resistant *A. fumigatus* (AR*Af*) isolates underwent confirmatory molecular identification. AR*Af sensu stricto* (*SS*) isolates were further analysed by *CYP51A* gene and promoter sequencing to detect resistance-associated mutations.

**Results:**

Most isolates (246/249; 99%) were identified as *A. fumigatus SS*, while three isolates were molecularly identified as *A. fumigatiaffinis*, *A. lentulus* and *A. pseudoviridinutans*. Amphotericin B and echinocandins demonstrated good *in vitro* activity against *A. fumigatus SS*, whereas posaconazole was the most active azole *in vitro*. Cryptic species exhibited reduced susceptibility compared with *A. fumigatus SS*. Two isolates (1%; *A. fumigatiaffinis* and *A. lentulus*) were amphotericin B-resistant. Five isolates (2%) were AR*Af*, including all three cryptic species (from patients with chronic pulmonary aspergillosis; one pan-AR*Af*) and two *A. fumigatus SS* strains. One AR*Af SS* isolate (from a cystic fibrosis patient) was pan-AR*Af* and harboured the TR_46_/Y121F/T289A *CYP51A* mutation, while the second (from an ICU patient) displayed resistance to itraconazole, isavuconazole and posaconazole without detectable *CYP51A* mutations.

**Conclusions:**

Although the clinical AR*Af* rate remains low in Greece, the first detection of pan-azole resistance associated with environmentally driven resistance mechanisms or cryptic species highlights the importance of surveillance, accurate species identification and routine AFST.

## Introduction

The emergence of azole-resistant *A. fumigatus* (AR*Af*) and cryptic species within *Aspergillus* section *Fumigati* poses a significant clinical challenge, as it is associated with increased mortality and therapeutic failure.^1,2^ Marked geographic heterogeneity in the prevalence of these species emphasizes the importance of regional epidemiological monitoring.^3^ In Greece, invasive aspergillosis affects an estimated 10% of haematological patients.^4^ Nevertheless, data on clinical *A. fumigatus* species complex (SC) isolates are scarce,^3^ and antifungal susceptibility testing (AFST) for filamentous fungi is not routinely performed,^5^ rendering the burden and molecular epidemiology of AR*Af* incompletely defined at the national level. A nationwide surveillance study on soil samples has shown the presence of the environmentally acquired TR_46_/Y121F/T289A resistance mechanism, associated with pan-azole resistance, in an *A. fumigatus sensu stricto* (*SS*) isolate.^6^ Whether such resistance patterns are present among patient-derived isolates remains unknown.

This multicentre study aimed to characterize the antifungal susceptibility of clinical *A. fumigatus* SC isolates in Greece, focusing on azole resistance profiles and underlying mechanisms, and to determine the presence of cryptic species.

## Materials and methods

### Isolate collection

This retrospective study included 249 *A. fumigatus* SC isolates from a multicentre collection representing six tertiary care hospitals across Greece (Athens, Thessaloniki and Heraklion; three University-affiliated and one paediatric) and the Mycology Reference Laboratory of the National and Kapodistrian University of Athens (Department of Microbiology, Medical School). Isolates dating from 2006 to 2025 were retrieved from existing clinical repositories, routinely archived when deemed clinically significant pathogens based on institutional diagnostic protocols, excluding obvious environmental or mucosal contaminants, and were obtained from individual patients, primarily from respiratory specimens [bronchoalveolar lavage (BAL), bronchial secretions, sputum, and tracheal aspirates] and sterile sites (tissue biopsies and pleural fluid). All stored non-duplicate isolates during the study period were included without pre-selection based on antifungal susceptibility profiles. The study focused on the microbiological and molecular characterization of isolates, with detailed patient-level clinical data collected only for AR*Af* cases, where available.

All isolates were transported to the central reference laboratory (‘Attikon’ University Hospital) and stored at −70°C in 10% glycerol (AppliChem, Darmstadt, Germany). Before analysis, isolates were revived by subculture on Sabouraud glucose agar supplemented with gentamicin and chloramphenicol (SGC; Oxoid, Athens, Greece) and incubated at 30°C for 5–7 days.

### Phenotypic identification

Preliminary identification was performed at each centre based on colony morphology and microscopic characteristics.^7^ All isolates were retrospectively re-evaluated at the central laboratory to confirm SC-level identification.^7^ To presumptively differentiate *A. fumigatus SS*, isolates were also subcultured on SGC and incubated at 48°C.^7^

### Susceptibility testing

Isolates were subjected to *in vitro* AFST following the EUCAST broth microdilution reference methodology (E.Def 9.4).^8^ For echinocandins, a modification of the E.Def 9.4 protocol using a lower inoculum (10^3^ instead of 10^5^ CFU/mL) was applied, based on a multicentre evaluation that demonstrated its reproducibility and defined an upper limit of the wild-type (WT) population (0.03 mg/L) for micafungin and anidulafungin.^9^ Antifungals tested included amphotericin B, itraconazole, posaconazole, isavuconazole (all from Sigma-Aldrich, Athens, Greece), voriconazole (Pfizer Ltd., Kent, UK), anidulafungin (Pfizer, Groton, CT, USA) and micafungin (Astellas Pharma, Tokyo, Japan). Drug concentrations ranged from 0.06–8 mg/L for amphotericin B and 0.008–8 mg/L for the remaining agents. Quality control strains (*A. flavus* ATCC 204304, *A. fumigatus* ATCC 204305, *Pichia kudriavzevii* ATCC 6258, and *Candida parapsilosis* ATCC 22019), together with a clinical TR_34_/L98H *A. fumigatus* isolate (V52–35; EUCAST minimum inhibitory concentrations (MICs) > 16 mg/L for itraconazole and 4 mg/L for voriconazole),^10^ were included. Plates were incubated for 48 h at 35 ± 2°C. MICs for amphotericin B and azoles were defined as the lowest concentration resulting in complete visible growth inhibition, while for echinocandins, the minimum effective concentration (MEC) was determined as the lowest concentration inducing hyphal morphological alterations. Resistant isolates per EUCAST clinical breakpoints for *A. fumigatus* SC (amphotericin B/itraconazole/voriconazole/isavuconazole >1 mg/L and posaconazole >0.125 mg/L) were retested.

### Molecular identification

Isolates not growing at 48°C or exhibiting reduced azole susceptibility were further analysed for molecular identification and resistance mechanism characterization. Species-level identification was performed by sequencing the ITS1-5.8S-ITS2 region and a partial β-tubulin gene fragment.^11^ Subsequently, the coding and promoter regions of the *CYP51A* gene were sequenced in AR*Af* isolates to detect potential azole resistance-associated genetic alterations.^12^

### Data analysis

MIC/MEC ranges, geometric mean (GM) MICs/MECs, MIC_50_/MEC_50_ and MIC_90_/MEC_90_ (concentrations inhibiting 50% and 90% of isolates) were determined for each agent. Cross-resistance was tested using Spearman correlation of log_2_-transformed azole MICs. Temporal trends in azole resistance rates across 5-year intervals were evaluated with the Chi-square test for trend. Two-tailed *P*-values <0.05 were considered statistically significant throughout. Statistical analyses were conducted with GraphPad Prism, version 9.0 (GraphPad Software, San Diego, CA, USA).

## Results

Most *A. fumigatus* SC isolates (246/249; 99%) grew at 48°C, consistent with identification as *A. fumigatus SS*. The remaining three isolates were molecularly identified as *A. fumigatiaffinis*, *A. lentulus* and *A. pseudoviridinutans*.

### MIC data

Antifungal susceptibility patterns are summarized in Table 1. Among *A. fumigatus SS*, amphotericin B demonstrated good *in vitro* activity (GM MIC 0.44 mg/L). Echinocandins were also active *in vitro* (anidulafungin GM MEC 0.009 mg/L; micafungin 0.012 mg/L). Among azoles, posaconazole displayed the highest *in vitro* activity (GM MIC 0.07 mg/L), followed by itraconazole (0.32 mg/L), voriconazole (0.57 mg/L) and isavuconazole (0.64 mg/L). Two isolates (2/246; 1%) were resistant to at least one azole. Overall, the three cryptic *A. fumigatus* isolates showed distinct susceptibility profiles with reduced susceptibility to azoles and amphotericin B compared with *A. fumigatus SS*, whereas echinocandins retained good *in vitro* activity (anidulafungin MECs ≤0.008 mg/L; micafungin MECs ≤0.03 mg/L).

**Table 1.** *In vitro* antifungal susceptibility profile of 249 clinical *A. fumigatus* species complex isolates determined with the EUCAST E.DEF 9.4^8^

Species (No of isolates)	Antifungal agent	Number of isolates with MIC (mg/L) of:											MIC_50_/MEC_50_	MIC_50_/MEC_90_	GM MIC/MEC	% Resistance
		≤0.008	0.016	0.03	0.06	0.125	0.25	0.5	1	2	4	≥8				
*A. fumigatus SS*	Amphotericin B					2	52	180	12				0.5	0.5	0.44	0
(*n* = 246)	Itraconazole				2	22	127	78	15			2*	0.25	0.5	0.32	1
	Voriconazole					2	25	144	74		1*		0.5	1	0.57	0.5
	Isavuconazole					2	9	135	98	1*	1*		0.5	1	0.64	1
	Posaconazole			12	153	79	1*	1*					0.06	0.125	0.07	1
	Anidulafungin	213	30	3									≤0.008	0.016	0.009	—
	Micafungin	109	128	9									0.016	0.016	0.012	—
*A. fumigatiaffinis*	Amphotericin B										1*		—	—	—	100
(*n* = 1)	Itraconazole							1					—	—	—	0
	Voriconazole									1*			—	—	—	100
	Isavuconazole									1*			—	—	—	100
	Posaconazole					1							—	—	—	0
	Anidulafungin	1											—	—	—	—
	Micafungin	1											—	—	—	—
*A. lentulus*	Amphotericin B											1*	—	—	—	100
(*n* = 1)	Itraconazole									1*			—	—	—	100
	Voriconazole										1*		—	—	—	100
	Isavuconazole								1				—	—	—	0
	Posaconazole					1							—	—	—	0
	Anidulafungin	1											—	—	—	—
	Micafungin	1											—	—	—	—
*A. pseudoviridinutans*	Amphotericin B								1				—	—	—	0
(*n* = 1)	Itraconazole											1*	—	—	—	100
	Voriconazole											1*	—	—	—	100
	Isavuconazole											1*	—	—	—	100
	Posaconazole						1*						—	—	—	100
	Anidulafungin	1											—	—	—	—
	Micafungin			1									—	—	—	—
Total	Amphotericin B					2	52	180	13		1*	1*	0.5	0.5	0.45	1
(*n* = 249)	Itraconazole				2	22	127	79	15	1*		3*	0.25	0.5	0.33	2
	Voriconazole					2	25	144	74	1*	2*	1*	0.5	1	0.59	2
	Isavuconazole					2	9	135	99	2*	1*	1*	0.5	1	0.66	2
	Posaconazole			12	153	81	2*	1*					0.06	0.125	0.08	1
	Anidulafungin	216	30	3									≤0.008	0.016	0.009	—
	Micafungin	111	128	10									0.016	0.016	0.012	—

Antifungal-resistant isolates based on EUCAST clinical breakpoints for *A. fumigatus* species complex (amphotericin B/itraconazole/voriconazole/isavuconazole > 1 mg/L; posaconazole > 0.125 mg/L) are indicated in bold and marked with an asterisk. For echinocandins, all isolates exhibited wild-type phenotypes (MECs ≤0.03 mg/L).^9^

MEC, minimum effective concentration; MIC, minimum inhibitory concentration; GM, geometric mean; *SS*, *sensu stricto*.

### Resistant isolates

Amphotericin B resistance was detected in 2/249 (1%) isolates, both belonging to cryptic species (*A. lentulus* and *A. fumigatiaffinis*), although species-specific breakpoints for cryptic species are lacking. Overall, 5/249 (2%) were AR*Af*, including all three cryptic isolates and two *A. fumigatus SS* strains. Among cryptic species, *A. lentulus* was voriconazole- and itraconazole-resistant, *A. fumigatiaffinis* voriconazole- and isavuconazole-resistant, while *A. pseudoviridinutans* displayed a pan-azole-resistant phenotype. All were recovered from BAL specimens of patients with chronic pulmonary aspergillosis (CPA), with no prior antifungal therapy. Regarding the AR*Af SS* isolates, one was a pan-azole-resistant TR_46_/Y121F/T289A *CYP51A* mutant, cultured in 2018 from the sputum of a 16-year-old cystic fibrosis (CF) patient without prior azole exposure. The second isolate, recovered from an ICU patient's pleural fluid, was resistant to itraconazole, isavuconazole and posaconazole but susceptible to voriconazole; no *CYP51A* mutations were detected and prior antifungal exposure was unknown (Table 1 and Table 2). Clinical outcomes were not available for patients with AR*Af* isolates. AR*Af* isolates were identified in three of the seven centres. Evaluating overall collection patterns across 5-year intervals (2006–2010: n = 48; 2011–2015: n = 38; 2016–2020: n = 95; 2021–2025: n = 68), azole resistance rates remained low (2.1%, 0%, 3.3% and 1.5%, respectively), with no statistically significant temporal trend (*P* = 0.89). Specifically, AR*Af* isolates were distributed sporadically over time, with no clear temporal clustering, comprising one ARA*f SS* without detectable *CYP51A* mutation in 2006, one TR_46_/Y121F/T289A ARA*f SS* in 2018, two AR*Af* cryptic in 2020, and one AR*Af* cryptic in 2025 (Figure 1). Although the % of AR*Af* doubled in the last 10 years from 1.17% in 2006–2015 to 2.52% in 2016–2025, this increase was not statistically significant (*P* = 0.48).

**Figure 1.**
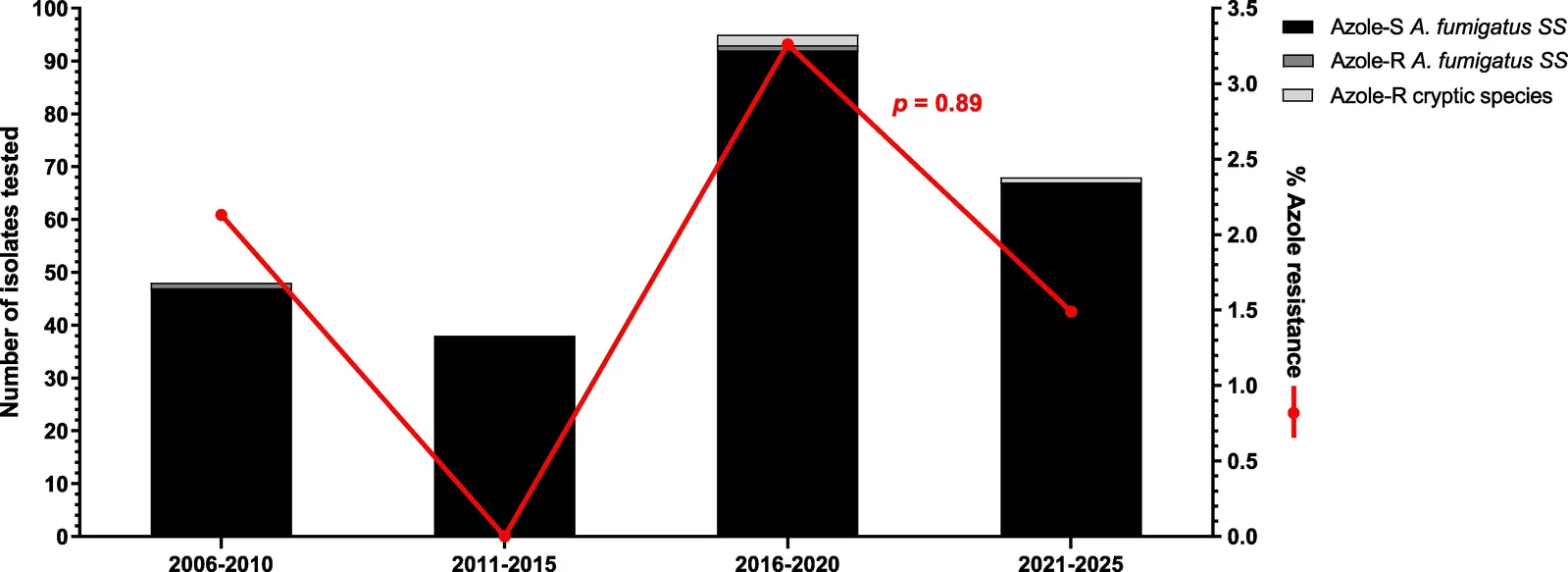
Temporal trends in isolate collection and azole resistance rates over 5-year intervals (2006–2025). Bars represent the total number of tested isolates per period, while the line indicates the azole resistance rate (right y-axis). No significant temporal trend in azole resistance was observed (*P* = 0.89). S, susceptible; *SS*, *sensu stricto*; R, resistant.

**Table 2.** Characteristics and antifungal susceptibility of azole-resistant clinical *A. fumigatus* species complex isolates

Species	Sample	Underlying condition	Previous use of azoles	*CYP51A* substitution	EUCAST MIC (mg/L)				
					AMB	ITC	VRC	ISA	POS
*A. fumigatiaffinis*	BAL	CPA	Νο	NP	4*	0.5	2*	2*	0.125
*A. lentulus*	BAL	CPA	Νο	NP	>8*	2*	4*	1	0.125
*A. pseudoviridinutans*	BAL	CPA	No	NP	1	>8*	8*	8*	2*
*A. fumigatus SS*	Sputum	Cystic fibrosis	No	TR_46_/Y121F/T289A	0.5	>8*	4*	4*	0.25*
*A. fumigatus SS*	Pleural fluid	ICU patient	Unknown	No	0.5	>8*	1	2*	0.5*

Antifungal-resistant isolates based on EUCAST clinical breakpoints for *A. fumigatus* species complex (AMB/ITC/VRC/ISA > 1 mg/L; POS > 0.125 mg/L) are indicated in bold and marked with an asterisk.

AMB, amphotericin B; BAL, bronchoalveolar lavage; CPA, chronic pulmonary aspergillosis; ICU, intensive care unit; ITC, itraconazole; ISA, isavuconazole; MIC, minimum inhibitory concentration; NP, not performed; POS, posaconazole; *SS*, *sensu stricto*; VRC, voriconazole.

### MIC correlation

Azole MICs among *A. fumigatus* SC isolates were positively correlated (*P* < 0.0001) (Figure 2). The strongest correlations were observed for itraconazole–posaconazole (Spearman r = 0.53), voriconazole–isavuconazole (r = 0.50) and itraconazole–isavuconazole (r = 0.48). Weak correlations were noted for isavuconazole–posaconazole (r = 0.34), voriconazole–posaconazole (r = 0.31) and voriconazole–itraconazole (r = 0.28).

**Figure 2.**
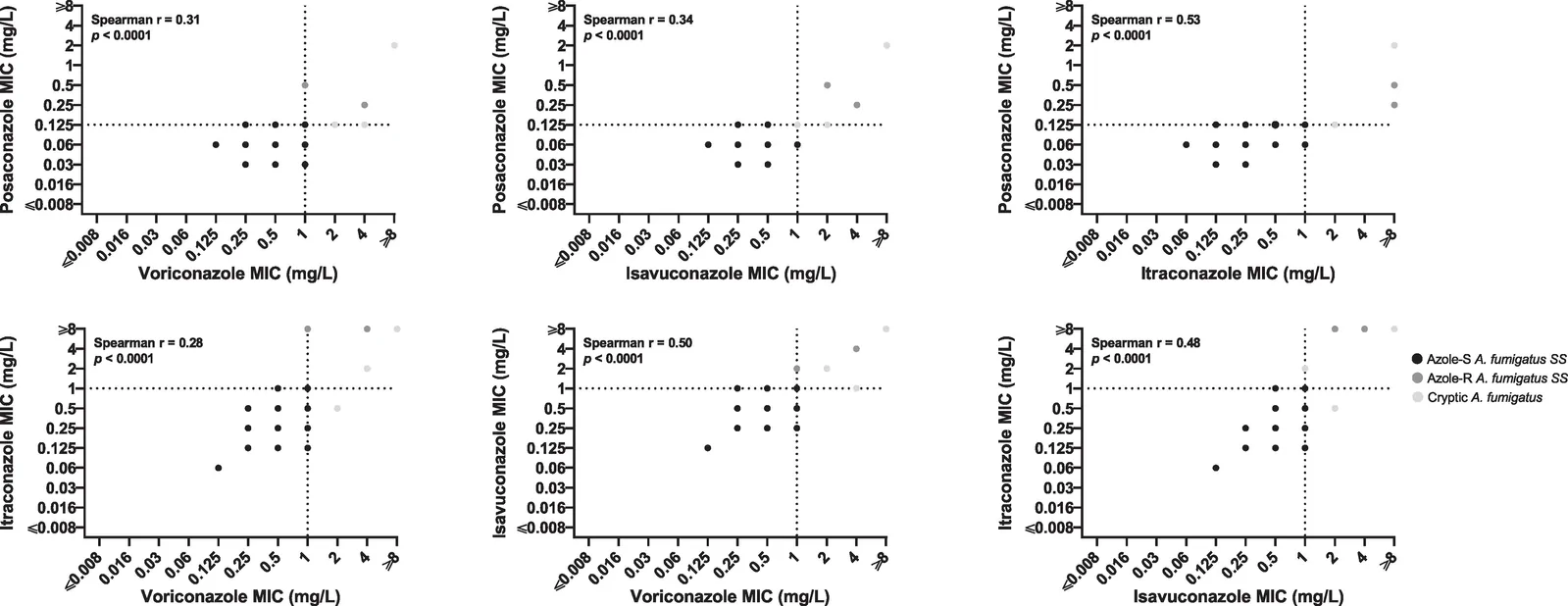
Distribution and correlation of azole MICs among clinical *A. fumigatus* species complex isolates. EUCAST clinical breakpoints for *A. fumigatus* species complex are indicated by black broken lines. Spearman correlation coefficients (r) and corresponding *P*-values are shown for each comparison. S, susceptible; *SS*, *sensu stricto*; R, resistant.

## Discussion

The prevalence of azole resistance (2%) and cryptic species (1%) in clinical isolates was found to be low in Greece. The three isolates identified as cryptic species (*A. fumigatiaffinis*, *A. lentulus* and *A. pseudoviridinutans*), all had reduced azole susceptibility. Additionally, two AR*Af* isolates were detected: one pan-azole-resistant carrying the environmentally associated TR_46_/Y121F/T289A *CYP51A* mutation, and one showing multi-azole resistance without *CYP51A* alterations. Although the % of AR*Af* isolates doubled in the last 10 years (from 1.17% in 2006–2015 to 2.52% in 2016–2025) mainly due to the emergence of azole-resistant cryptic species, these strains appeared sporadically with no apparent temporal clustering or significant upward trend, suggesting limited emergence within the clinical setting. However, given the relatively low number of isolates per year, it remains difficult to detect smaller temporal trends or changes in resistance rates. Amphotericin B and echinocandins retained good *in vitro* activity, with amphotericin B resistance limited to two cryptic species (overall 1%).

In Greece, antifungal resistance in clinical *A. fumigatus* SC remains poorly characterized.^3^ In a single-centre collection, none of 29 *A. fumigatus* SC isolates obtained during 2009–2011 (screening with 4-well azole-supplemented plates)^13^ and in 2020 (EUCAST methodology)^14^ were AR*Af*. Similarly, six isolates from a multicentre study in Northern Greece (2020–2022) exhibited amphotericin B and azole WT phenotypes, as determined by gradient concentration strips.^15^ Conversely, earlier multicentre data (1998–2009) using CLSI methodology reported elevated itraconazole and posaconazole MICs falling outside the WT distribution in 6/42 (14%; five *A. fumigatus SS* and one cryptic species; MICs 2 mg/L) and 3/42 (7%; all *A. fumigatus SS*; MICs 1–2 mg/L) isolates, respectively, while voriconazole resistance was not observed (MICs 0.03–1 mg/L).^16^ Nevertheless, the absence of molecular verification of underlying resistance mechanisms in those strains should be acknowledged. In the same study, 4/42 (10%) isolates (two *A. fumigatus SS* and two cryptic species) displayed non-WT amphotericin B MICs (4–16 mg/L).^16^ Overall, these scattered reports preclude definitive conclusions on the contemporary epidemiological landscape.

Within this context, this study provides a comprehensive snapshot of antifungal susceptibility among clinical *A. fumigatus* SC isolates in Greece, spanning two decades (2006–2025). The population was dominated by *A. fumigatus* SS (99%), mirroring global surveillance data (96%–99%).^17–20^ While cryptic species were identified at a low frequency (1%), in line with their reported rarity in clinical settings,^17–19^ their detection represents an emerging challenge that should not be overlooked in diagnostic algorithms. Here, all three cryptic isolates exhibited elevated azole MICs, including one pan-azole-resistant, whereas two showed concomitantly elevated amphotericin B MICs, consistent with previous reports.^17^ However, as EUCAST breakpoints are driven by the overwhelming dominance of *A. fumigatus SS* in surveillance datasets, applying these thresholds to cryptic species requires cautious interpretation in the absence of species-specific criteria. This underscores the importance of accurate species-level identification in clinically relevant lower respiratory tract samples. Since cryptic species are morphologically indistinguishable from *A. fumigatus SS*, they may remain undetected by routine phenotypic methods. Indeed, while they typically represent <3% of all *Aspergillus* section *Fumigati* clinical isolates, they can account for 10%–30% of AR*Af* strains morphologically misidentified as *A. fumigatus SS.*^21^ Thus, the correct species identification is a clinical necessity for guiding tailored antifungal therapy. Molecular approaches are considered the gold standard, but they are not widely available or easily performed in a routine setting. MALDI-TOF MS offers a practical alternative, yet misidentification of some cryptic species remains a challenge.^22^

The detection of acquired azole resistance mechanisms in *A. fumigatus SS* further highlights Greece’s dynamic epidemiology. To our knowledge, this constitutes the first clinical report of TR_46_/Y121F/T289A-mediated resistance in Greece, identified in 2018 in a pan-azole-resistant isolate recovered from the sputum of an azole-naive CF patient. This finding aligns with our previous environmental investigations, which in 2017 identified the same TR_46_ genotype and similar phenotype (MIC >8 mg/L for itraconazole voriconazole and isavuconazole and 1 mg/L for posaconazole) in an *A. fumigatus SS* isolate.^6^ While a direct epidemiological link cannot be formally established, the close temporal and molecular proximity suggests an environmental acquisition. Of note, CF patients may be particularly susceptible to colonization and subsequent infection by AR*Af* owing to their altered airway microenvironment and underlying local immune deficiency.^23^ Accordingly, the recovery of a strain harbouring the TR_46_/Y121F/T289A resistance allele from sputum may reflect early airway colonization by an environmentally acquired resistant strain. Meanwhile, its presence in an azole-naive patient may indicate its environmental origin, as this tandem-repeat-mediated mechanism is typically associated with the off-site selection of resistance through agricultural triazole use.^24^ Interestingly, this observation appears to reinforce our hypothesis regarding the lag time between regional azole fungicide registration and the emergence of environmentally driven clinical resistance.^6^ Given that tebuconazole and propiconazole were registered in Greece in the late 2000s, the detection of the first clinical TR_46_ isolate in 2018 is consistent with the anticipated 10-year resistance window, echoing trends recorded in the Netherlands.^25^ Since TR_46_/Y121F/T289A is commonly associated with high-level resistance across multiple medical triazoles,^24^ this sentinel case underscores the need for vigilance and a One Health framework integrating clinical and environmental surveillance. Notably, the globally predominant TR_34_/L98H environmental allele was not detected in our cohort and has never been documented in Greek clinical or environmental isolates to date.^3^ This geographic variation may reflect local differences in agricultural triazole usage patterns or distinct environmental dynamics.

Beyond the aforementioned environmental genotype, no *CYP51A* point mutations associated with long-term clinical azole therapy were detected in our cohort. Among the AR*Af* cases for which clinical data were available, none of the patients had documented prior azole exposure, consistent with an environmental rather than a treatment-driven origin. However, given the lack of longitudinal clinical records for the susceptible cohort, the true rate of clinical azole exposure across the entire population cannot be definitively evaluated. In fact, the detection of an *ARAf SS* isolate lacking *CYP51A* alterations highlights alternative resistance pathways. This isolate exhibited resistance to multiple azoles while remaining susceptible to voriconazole, a phenotype frequently ascribed to non-*CYP51A*-mediated mechanisms like efflux pump overexpression or mutations in other ergosterol biosynthesis genes.^24^ Such cases may pose a significant diagnostic challenge in the common scenario of culture-negative aspergillosis, as resistance screening directly from clinical specimens with molecular assays targeting only the ‘hot-spot’ *CYP51A* mutations would erroneously flag them as azole-susceptible, potentially leading to inappropriate empirical therapy. This complexity is further compounded by the variable azole cross-resistance patterns. While significant positive correlations among certain azole MICs, particularly for the itraconazole–posaconazole and voriconazole–isavuconazole pairs, reflecting expected structural similarities, were recorded, the weaker correlations and divergent susceptibility profiles noted in other instances underscore the unpredictability of resistance phenotypes, as previously described.^26^ Collectively, these observations strengthen the necessity of routine phenotypic AFST to guide individualized therapy.

Overall, the clinical AR*Af* prevalence was low (2%), positioning Greece at the lower end of Southern European surveillance data (7%),^17,19^ and mirroring trends from multicentre studies conducted in Turkey (3%)^27^ and Australia (2%).^28^ In fact, azole resistance remained consistently low, with no statistically significant temporal trend in our country, unlike the Netherlands, where AR*Af* in clinical settings has surged in recent years.^29^ Moreover, this low detection frequency contrasts with rates ranging above 20% and as high as 52% in certain high-risk cohorts.^30,31^ Indeed, our findings suggest that azole-resistant phenotypes may primarily affect vulnerable groups, such as CPA and CF patients, underscoring the need for active surveillance programs including routine resistance screening and the avoidance of known high-risk environmental exposures in these populations. Notably, while patient-to-patient transmission of AR*Af* strains is highly unlikely in the setting of IA, it remains a recognized possibility among colonized CF individuals.^32^ Regarding polyenes, amphotericin B resistance was rare (1%) and exclusively associated with cryptic species, in line with the generally preserved activity of this agent against *A. fumigatus SS*^24^ and previous reports indicating that elevated MICs within the section *Fumigati* were strictly linked to specific cryptic taxa.^17^

This study has limitations, including its retrospective, laboratory-based multicentre design spanning two decades. Thus, the reported 2% azole-resistance rate should be interpreted as the proportion observed in this collection rather than a definitive national prevalence. The lack of electronic medical record systems across Greek hospitals^33^ restricted the retrieval of comprehensive longitudinal clinical records for all cases; detailed patient-level characteristics could only be obtained for AR*Af* cases, and hence, the interpretation of overall resistance dynamics and their clinical impact should be made with caution. Additionally, the retrospective use of laboratory repositories introduces potential selection bias, as isolate archiving relied on local laboratory practices and clinical evaluation of significance at the time of isolation, rather than a prospective, standardized surveillance protocol. Furthermore, while geographical coverage does not encompass all rural provinces, it focused on the most densely populated regions where tertiary healthcare services (where most of azole prophylaxis is administered) are concentrated, serving over 53% of the Greek population. Finally, molecular species identification was performed only in isolates unable to grow at 48°C or showing reduced azole susceptibility and therefore some thermotolerant susceptible cryptic species may have been missed. Nonetheless, it represents the most comprehensive multicentre evaluation of clinical *A. fumigatus* SC resistance in Greece to date. By building upon our previous environmental investigations,^6^ it contributes to a more integrated mapping of the resistance landscape in the region. Moreover, the use of standardized EUCAST AFST methodology enables direct comparison with European surveillance data. Spanning an extended time period and incorporating both phenotypic and molecular characterization, our data provide a critical baseline for longitudinal surveillance. In parallel, they serve as a sentinel warning that the clinical landscape is no longer static; the detection of TR_46_-mediated resistance and the presence of cryptic species signal the need for a national registry or a structured One Health monitoring system to optimize antifungal stewardship, ensure the selection of appropriate therapeutic regimens, and preserve the long-term efficacy of the limited antifungal armamentarium available against aspergillosis.

Taken together, this multicentre study provides the first long-term overview of clinical *A. fumigatus* SC susceptibility in Greece, marking the clinical emergence of the TR_46_/Y121F/T289A lineage. While the low AR*Af* prevalence is reassuring, the presence of environmentally driven mechanisms and azole-resistant cryptic species highlight the evolving and multifaceted nature of resistance. High-level vigilance, the integration of routine AFST and molecular diagnostics into clinical workflows, particularly for high-risk cohorts, and coordinated clinical and environmental surveillance are vital to elucidate the epidemiology of this emerging airborne threat at a nationwide scale.
